# *Klebsiella variicola* Infection in a Second Trimester Twin Pregnancy: An Underreported Cause of Chorioamnionitis

**DOI:** 10.3390/diagnostics15040480

**Published:** 2025-02-17

**Authors:** Maria Paola Bonasoni, Alice Ferretti, Immacolata Blasi, Giuseppina Comitini, Lorenzo Aguzzoli, Marcellino Bardaro, Giuseppe Russello, Edoardo Carretto

**Affiliations:** 1 Pathology Unit, Azienda USL-IRCCS di Reggio Emilia, 42122 Reggio Emilia, Italy; 2 Unit of Obstetrics and Gynecologic Oncology, Azienda USL-IRCCS di Reggio Emilia, 42122 Reggio Emilia, Italy; alice.ferretti@ausl.re.it (A.F.); immacolata.blasi@ausl.re.it (I.B.); giuseppina.comitini@ausl.re.it (G.C.); lorenzo.aguzzoli@ausl.re.it (L.A.); 3 Clinical Microbiology Laboratory, Azienda USL-IRCCS di Reggio Emilia, 42122 Reggio Emilia, Italy; marcellino.bardaro@ausl.re.it (M.B.); giuseppe.russello@ausl.re.it (G.R.); edoardo.carretto@ausl.re.it (E.C.)

**Keywords:** *Klebsiella variicola*, twin pregnancy, chorioamnionitis

## Abstract

**Background and Clinical Significance:** *Klebisella variicola* belongs to the *Klebsiella pneumoniae* complex. It is a Gram-negative, facultative anaerobic, and nonmotile bacillus, mainly isolated in plants. However, as an emerging human pathogen, it has been isolated in immunocompromised patients with urinary tract infections, pneumonia, and bacteremia. *K. variicola* infection in pregnancy, responsible for acute chorioamnionitis, has never been reported. **Case Presentation:** We present a case of a twin pregnancy at 17 + 5 weeks in which chorioamnionitis and fetal inflammatory responses such as funisitis and chorionic vasculitis were due to an ascending infection of *K. variicola*. The pathogen was isolated postmortem in fetal blood and tissues and the placenta using MALDI-ToF mass spectrometry (MALDI-ToF MS). The accuracy of this microbiological diagnosis sheds further light on the epidemiology and virulence of *K. variicola* in the prenatal setting. **Conclusions:** In the case of miscarriage, microbiological investigations on the fetus should always be recommended to identify the exact microorganism in order to target the medical treatment and manage subsequent pregnancies.

## 1. Introduction

The family *Enterobacteriaceae* comprises the *Klebsiella* genus, which further includes the *Klebsiella pneumoniae* complex. Phylogenetic variety within *K. pneumoniae* clinical isolates was demonstrated by analyzing *rpoB* and *gyrB* genes. The investigation detected six separate *K. pneumoniae* phylogroups designated *K. pneumoniae* (Kp1), K. *quasipneumoniae* [subsp. *quasipneumoniae* (Kp2), subsp. *similipneumoniae* (Kp4)], *K. variicola* (Kp3), and two unnamed phylogroups (Kp5 and Kp6) [1]. Kp3 was identified as *K. variicola*, a novel bacterial species, after biochemical and genetic tests on plants and clinical isolates. The term “variicola” derives from the Latin word va.ri.i’co.la., which means “inhabitant of different places” [2]. *K. variicola* is a Gram-negative, facultative anaerobic, and nonmotile bacillus. It has been mainly isolated in vegetal ecosystems in which it supports nitrogen fixation and plant thriving. Plants are considered a biological reservoir of *K. variicola,* which is a regular endophyte and exceptionally a pathogen. For example, a natural niche is represented by the successful symbiosis between *K. variicola* and leaf-cutter ant colonies, which obtain their nitrogen necessities from this microorganism [3].

On the other hand, infections in humans and wild and farm animals, especially bovines, have also been observed [3]. In humans, *K. variicola* has often been isolated from bloodstream infections (BSIs), respiratory infections, and urinary tract infections (UTIs) [3]. *K. variicola* is phenotypically indistinguishable from *K. pneumoniae*, and, therefore, the epidemiology of this microorganism was not fully elucidated in human microbiology until mass spectrometry (MALDI-ToF) was not routinely used. Although with some degree of inaccuracy, MALDI-ToF-MS is able to distinguish among the *K. pneumoniae* complex and allow the identification of *K. variicola*, even if molecular methods remain the gold standard for the correct speciation of these microorganisms [3]. The possibility of identifying *K. variicola* in clinical samples can shed light on its epidemiology, virulence, and clinical relevance.

Among the *K. pneumoniae* complex, in pregnancy, *K. pneumoniae* represents an underreported cause of acute chorioamnionitis (AC) and miscarriage, with few cases described [4,5,6,7,8]. To the best of our knowledge, intrauterine fetal death (IUFD) due to the *K. variicola* phylogroup has never been described. Herein, we report a case of a twin pregnancy that resulted in second-trimester chorioamnionitis at 17 weeks + 5 days of gestation. The bacterium was isolated in fetal blood, fetal tissues, and placental cultures. Maternal clinical history and postmortem findings are also presented and discussed.

## 2. Case Presentation

The mother was a 36-year-old woman, gravida 2, para 0, presenting a dizygotic dichorionic–diamniotic twin pregnancy with separate placental discs, derived from a homologous in vitro fertilization. Two years before the pregnancy, the patient had a spontaneous abortion at 20 weeks of gestational age, due to cervical incompetence. The medical clinical history was otherwise unremarkable. In the current pregnancy, at 16 weeks gestation, a cervical cerclage was placed due to cervical incompetence. At 16 weeks + 5 days the patient was admitted for preterm premature rupture of membranes (PPROM) of twin 1, as at ultrasound (US), the amniotic fluid volume was overall reduced. US identified twin 1 as male, in cephalic presentation, located close to the cervical internal os, on the lower left side of the uterus. Twin 2, a female, with regular amniotic fluid volume, was in breech presentation, on the upper right side of the uterus. The cervical cerclage was regularly placed. Maternal white blood cells were slightly elevated (10.81x 1000/µL; normal value 4.00–10.00x 1000/µL). C-reactive protein (CRP) was within normal limits (0.36 mg/dL; normal value 0.00–0.50 mg/dL). Antibiotic treatment was started according to PPROM guidelines: intravenous ampicillin (2 g/6 h for 2 days), oral azithromycin 500 mg (single dose), then replaced by oral amoxicillin 500 mg once a day for 5 days [9,10]. After 5 days, the patient was discharged with careful observational planning. However, after 2 days, at 17 weeks + 5 days, she was admitted again with hyperpyrexia (39 °C), leukocytosis (21.48x 1000/µL), and high CRP (7.76 mg/dL). US results showed twin 1 with anhydramnios. Due to an overall poor fetal prognosis, the parents opted for legal termination of the pregnancy, and after delivery, both twins were sent for pathological examination.

**Figure 1:**
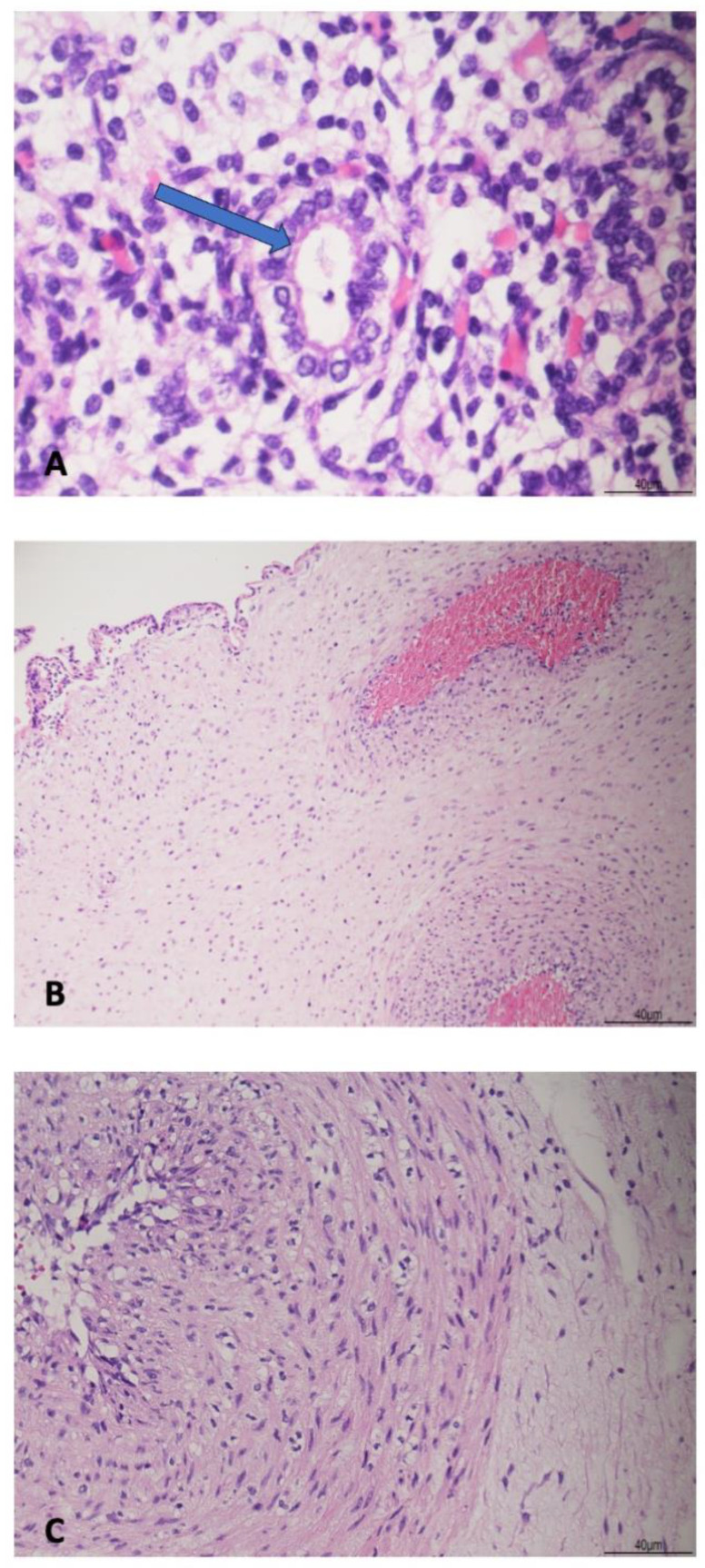
Fetal and placental infection of twin 1. (**A**): a pulmonary alveolus with a granulocyte and few bacilli (arrow) (HE staining 60×). (**B**): acute chorioamnionitis with neutrophils within the chorionic plate (HE staining 10×). (**C**): funisitis, one artery with intramural granulocytes extending to the Wharton’s jelly (HE staining 20×).

At autopsy, twin 1 was a non-macerated male fetus weighing 156 g and measuring 19 cm in crown–heel length. The other measurements were as follows: crown–rump length, 13 cm; foot length, 2.2 cm; head, chest, and abdominal circumference, 15 cm, 12.5 cm, and 12 cm, respectively. Anthropometric measurements were more compatible with 17 weeks gestation [11]. Grossly, the fetus was normal with no dysmorphic features. A mild palpebral and nuchal edema was observed. Internal examination disclosed mild pleural and abdominal serosanguinous effusions, but no anatomical anomalies. Histologically, few granulocytes were observed within the respiratory tract, occasionally associated with bacteria (Figure 1A). Some erythroblasts were found within the fetal vessels in most of the organs. The lungs were in the canalicular stage of development, as expected for gestational age [12]. The other organs showed no histological abnormalities.

The placenta was a single complete disc, weighing 70 g and measuring 12 × 8 × 2 cm. The membranes were diffusely opaque. Microscopically, there was acute chorioamnionitis (AC), with clusters of neutrophils extending from the chorionic plate to the sub-amniotic space (maternal inflammatory response stage 2/4, grade 1/2) (Figure 1B). The cord showed granulocyte infiltration of the three vessels with extension to the Wharton’s jelly (fetal inflammatory response stage 2/3, grade 1/2) (Figure 1C). 

Chorionic vasculitis was also present [13]. The placental parenchyma showed edematose mesenchymal villi. In the decidua, most of the arteries showed smooth muscle in the wall.

**Figure 2:**
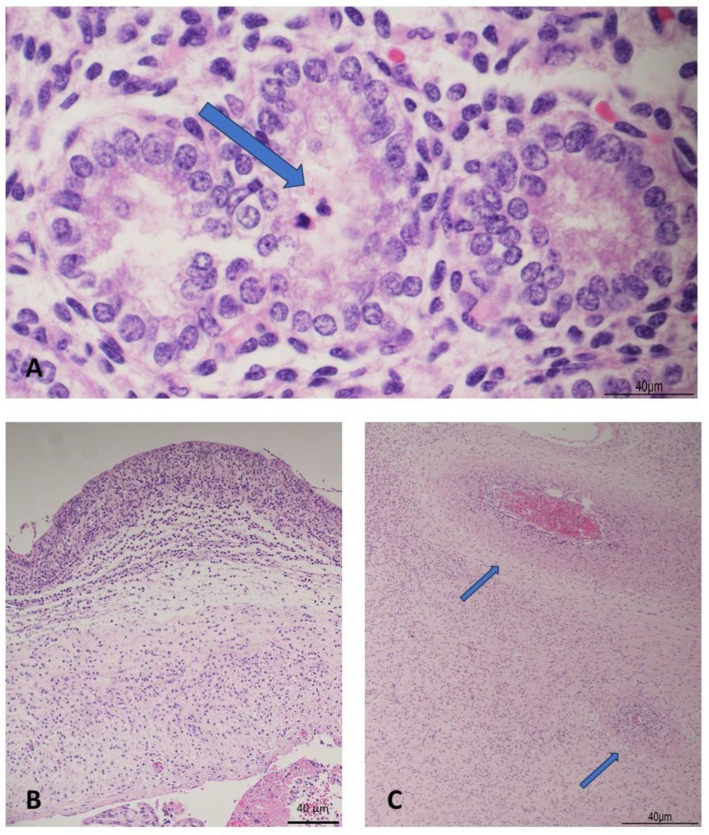
Fetal and placental infection of twin 1. (**A**): pulmonary alveoli with intraluminal neutrophils (arrow) (HE staining 60×). (**B**): severe chorioamnionitis (HE staining 10×). (**C**): chorionic vasculitis (arrows) (HE staining 4×).

Twin 2 was a non-macerated female fetus that weighed 150 g and measured 20 cm in crown–heel length. Anthropometric measurements, even in this baby, fitted more with 17 weeks gestation: crown–rump length 14 cm; foot length 2.6 cm; head, chest, and abdominal circumference 15 cm, 13 cm, and 12 cm, respectively [11]. External examination revealed mild palpebral and nuchal edema, but no other anomalies. Internally, mild pleural and abdominal serosanguinous effusions were the only relevant findings. Microscopically, some granulocytes were found within the respiratory airways, occasionally associated with bacteria (Figure 2A). As in twin 1, the lungs presented regular maturation for gestational age, with canalicular features [12]. The other organs were overall normal.

The single placenta weighed 40 g and measured 8 × 3 × 3 cm. The membranes were yellowish. At histology, AC was severe, with amnion necrosis (stage 4/4 and grade 2/2) (Figure 2B). Chorionic vasculitis, as a fetal inflammatory response, was also associated (stage 1/3, grade 1/2) [11]. In the placental parenchyma, clusters of villi were edematous. Most of the decidual arteries were not physiologically modified, with the preservation of the smooth muscle in the wall.

In both twins, fetal blood and tissues (lung and liver) and endogastric swabs, as well as subamniotic placental swabs and placental subchorionic samples were sent to the microbiology laboratory. All the samples were then processed according to standard procedures. In particular, the different specimens were inoculated on Columbia blood agar (CBA, sheep, 5%), chocolate agar (CA), and selective media (McConkey agar and mannitol salt agar—McC and MSA). For anaerobe detection, the samples were cultured on Schaedler agar (SCH). CBA, McC, and MSA were set at 36 °C in an aerobic atmosphere. The CA was incubated at 36 °C in microaerophilic conditions and SCH at 36 °C in an anaerobic atmosphere. All the different cultures were evaluated daily for three days. Identification and antimicrobial susceptibility testing were performed on all the isolates grown. To identify isolates, the MALDI-ToF MS (MALDI Biotyper, Bruker Daltonik, Germany), was used according to the manufacturer’s protocols. The instrument software was the MBT Compass IVD (n. 1832771), and the library was the MBT Compass Library 2023, covering 4239 species/entries and 12,299 microbial spectra. For susceptibility testing, the automated system Phoenix 100™ (Becton Dickinson, USA) was used, together with the agar diffusion method. All the samples yielded Gram-negative bacteria, identified using mass spectrometry as *K. variicola* with a score value (SV) > 2.3 for all the samples. This SV is generated by comparing the sample’s mass spectrum to a database of reference spectra and, in general, an SV ≥ 2.0 implies a high confidence identification with an identification at the species level. Upon evaluating the matches with the library microbial spectra for all the isolates, no matches with *K. pneumoniae* reference strains were present. The isolate’s susceptibility profile revealed a wild-type phenotype, being susceptible to all the antibiotics tested, except for the intrinsic resistances (ampicillin, amoxicillin, and ticarcillin). No ESBL, AmpC, or carbapenemase resistance traits were documented. Moreover, all the isolates did not show the hypermucoviscosity (HMV) phenomenon, since no isolate showed a positive “string test”, considering this test positive when a bacteriology inoculation loop or needle is able to generate a viscous string > 5 mm in length by stretching bacterial colonies on an agar plate.

According to the isolation of *K. variicola* in fetal blood, fetal tissues, and placenta, the mother underwent an endometrial biopsy three months after the miscarriage, and the microbiological cultures on it resulted as negative. No vaginal or rectal swabs were performed either at the time of miscarriage or after.

## 3. Discussion

Intrauterine infection is one of the main causes of miscarriage in the second trimester. They are usually ascending infections in which the pathogenetic agent comes from the genital tract, spreading from the vagina through the cervical canal and then infecting the amniotic fluid and the fetus. Hematogenous dissemination from maternal blood is less frequent, and the most common agents are *Listeria monocytogenes*, *Fusobacterium nucleatum*, viruses, and protozoa [14,15]. They first affect the placental villi and then the fetus.

In mid-pregnancy loss, a placental sign of infection is represented by histological AC. Fetal inflammatory responses such as funisitis and chorionic vasculitis can also be present, especially in the late second trimester, and in longstanding infection. In the fetus, neutrophils in the lungs and/or in the gastrointestinal tract mean active swallowing, supporting the evidence that infection preceded fetal death. The overall intrauterine inflammation triggers labor with PPROM and miscarriage. In the case of ascending infection, the pathogen agents, concentrated in the membranes and/or in the amniotic fluid, produce endotoxins and exotoxins, inducing the secretion of cytokines (tumor necrosis factor-α, interleukin-1a, interleukin-1b, and interleukins 6 and 8), arising a maternal and fetal inflammatory reaction. Then, the production and secretion of prostaglandins, leukocyte chemotactic substances, and metalloproteinase occur. In the end, the overall intrauterine inflammation triggers labor with PPROM and miscarriage [15,16]. In the third trimester, stillbirth due to infectious causes is uncommon, but the reason is still unclear, probably resulting from a balance between the more mature fetal immune system and the modification of the local microflora during pregnancy [15,17].

In ascending infections, AC is commonly caused by group B *Streptococcus, Peptostreptococcus*, *Escherichia coli*, *Bacteroides* species, *Ureaplasma urealyticum*, *Mycoplasma hominis, Enetrococcus* spp., and *Haemopphylus influenzae* [15]. *Listeria monocytogenes* and *F. nucleatum*, as already mentioned, derive from the maternal hematogeneous spread [18,19]. In case of second-trimester miscarriage, perinatal autopsy protocols recommend microbiological cultures on fetal blood and tissues, as well as placental subamniotic swabs and parenchymal cultures [20,21]. Indeed, the accurate identification of the microorganism can be fundamental in post-partum medical treatment, as therapy can be appropriately adjusted [7].

However, from a clinical perspective, the definition of AC is not appropriate and must be limited to the histopathological findings of neutrophils within the amniochorial membranes and/or umbilical cord. The notion of “Triple I” is more correct in identifying “Intrauterine Inflammation or Infection or both” [22,23,24]. This new concept has been introduced to evaluate more accurately fetal/neonatal outcomes, reducing morbidity and avoiding antibiotic overtreatment. Triple I diagnosis is made in the presence of maternal fever with one or more of the following: (1) fetal tachycardia (>160 bpm for 10 min or longer); (2) maternal white blood cells (WBCs) > 15,000 without corticosteroid; (3) purulent discharge from the cervix or detected visually on speculum; (4) biochemical or microbiologic amniotic fluid examination confirming amniotic infection. Triple I is then classified as “confirmed” or “suspected”. Triple I certainty requires either confirmation of amniotic fluid infection (Gram stain positivity for bacteria, low levels of glucose, high WBC without a bloody tap, positive microbiological cultures) or placental findings of acute inflammation (e.g., chorioamnionitis and/or funisitis). If these criteria are not properly met, triple I is categorized as “suspected” or “isolated maternal fever”, the latter reassessed as “not Triple I” [23]. Antibiotic therapy depends on Triple I characteristics, but a wide-spectrum treatment with ampicillin and gentamicin is usually administered. In the case of cesarean section, clindamycin or metronidazole should also be included to cover anaerobic bacteria [23].

In the case we described, *K. variicola* was found as the microorganism responsible for AC in a second-trimester twin pregnancy at 17 weeks + 5 days.

*K. variicola* belongs to the *K. pneumoniae* complex, which also includes seven related species. It contains *K. pneumoniae*, *K. quasipneumoniae* subsp. *quasipneumoniae*, *K. quasipneumoniae* subsp. *similipneumoniae*, *K. variicola* subsp. *variicola* (defined as *K. variicola*), *K. variicola* subsp. *Tropicalensis, K. africanensis* bacterial species, and *K. quasivariicola*, which is still under validation [3]. *K. variicola* typically grows in vegetal ecosystems, but pathogenicity in humans has been described, especially in immunocompromised patients with urinary tract infections, pneumonia, and bacteremia. Additional adverse conditions such as systemic lupus erythematous, neoplasia, diabetes mellitus, hepatobiliary diseases, solid organ transplantation, and alcoholism may worsen *K. variicola* infection [3].

In adults, *K. variicola* bloodstream infections have been described as highly virulent with a high incidence of mortality [25,26]. In neonates, hospital outbreaks have been scarcely reported but have been highly fatal [26,27]. In one study by Farzana et al. [26], the high mortality was associated with the acquired antimicrobial resistance genes *bla*NDM-1 and *bla*CTX-M-15, other than the virulence genes shared with *K. pneumonia.*

*K. variicola* displays four major virulence features: capsule, lipopolysaccharide (LPS), siderophores, and fimbriae. The main related genes are integrated into the chromosomes, but some hypervirulent strains may carry virulence genes in large plasmids [3].

In pregnancy, to the best of our knowledge, *K. variicola* has never been described, and therefore its epidemiology and virulence are unknown. On the whole, this underreporting of *K. variicola* infection may be attributed to the misidentification of *K. variicola* as *K. pneumoniae*. In our specific case, MALDI-ToF-MS was able to extrapolate the identification of *K. variicola* among the *K. pneumoniae* complex. However, the bacterium was not confirmed with molecular biology, which still remains the high standard method for its recognition. However, as in this case, *K. variicola* can also be distinguished using phenotypic methods. Conventional biochemical tests, such as the Voges–Proskauer test, citrate utilization, and sugar fermentation profiles, can help differentiate *K. variicola* from other members of the *K. pneumoniae* complex. Additionally, *K. variicola* exhibits distinct metabolic traits, including its ability to utilize specific carbon sources such as melezitose and raffinose, which can serve as key differentiators. Moreover, in our case, all the analyses performed with the MALDI-ToF methodology resulted in spectra with high scores (more than 2.4) without matches with other *K. pneumoniae* complex isolates. 

The correct identification of *K. variicola* can be paramount in widening the knowledge of its pathogenicity in humans. In our case, the bacterium was found in a twin pregnancy, in both siblings and placentas. In particular, *K. variicola* was isolated from fetal blood and tissues (lung and liver), endogastric swabs, as well as subamniotic placental swabs and placental subchorionic samples. Since the presence of chorioamnionitis and fetal inflammatory response (funisitis in twin 1 and chorionic vasculitis in twin 2, respectively) and few granulocytes in fetal lungs, *K. variicola* was undoubtedly the etiopathogenic agent.

Twin 1 was the closest to the cervix, located on the lower left side of the uterus, and the first exposed to the ascending infection, as it suffered from PPROM with amniotic fluid leakage. At postmortem, in the placenta, AC was stage 2/4 and grade 1/2. However, fetal inflammatory response (FIR) was intense with funisitis of the three vessels extended to the Wharton’s jelly (stage 2/3, grade 1/2) and chorionic vasculitis. Twin 2 was located on the upper right side of the uterus. In this fetus, maternal inflammatory response (MIR) involved the whole chorionic plate with amnion necrosis (stage 4/4, grade 1/2), but FIR was limited to the chorionic vessels (stage 1/3, grade 1/2). This difference in MIR and FIR may be explained as a diverse exposition of the fetuses to the bacterial load from ascending infection. Moreover, the mother underwent antibiotic therapy for 5 days before the induced delivery, and this factor should also be taken into account. However, twin 1 presented a more extended FIR, probably as a consequence of a more longstanding infection. It has been widely reported that FIR can develop as early as 17 weeks of gestation, though its effectiveness, related to the maturity of the fetal immune system, is unclear [16]. In both twins, granulocytes were found within the respiratory tract. The presence of neutrophils in the pulmonary airways can both be the consequence of aspiration of infected amniotic fluid and/or the intrinsic immune response against a pathogen. Even second-trimester fetuses are adequately equipped for external microorganisms and are able to recruit endogenous leukocytes. In fetal lungs, Scott et al. found in 5 fetuses the Y chromosome in alveolar neutrophils, demonstrating FIR [28].

In the case we described, *K. variicola* infection might have spread from the intestine.

*K. variicola* is a known resident of gut microbiota, as the other bacteria of the *K. pneumoniae* complex [29]. As part of the normal intestinal microbiome, it can colonize the vagina, similarly to *E. coli* and *K. pnuemoniae*, resulting in ascending infection and miscarriage [30,31].

As far as we are aware, the case we described is the first in pregnancy, in which *K. variicola* infection was thoroughly isolated in fetal blood and tissues, and placenta. In miscarriages, and especially in the suspect of infection, microbiological cultures on fetal and placental samples as well as maternal investigations should be highly recommended in order to identify the microorganism. This can be helpful in defining the epidemiology and the potential reservoirs and niches of replication, thus reducing the risk of recurrence in further pregnancies.

## Data Availability

The data presented in this study are available on request from the corresponding author.
